# PDL regeneration via cell homing in delayed replantation of avulsed teeth

**DOI:** 10.1186/s12967-015-0719-2

**Published:** 2015-11-14

**Authors:** Wenting Zhu, Qian Zhang, Yang Zhang, Lian Cen, Jun Wang

**Affiliations:** Department of Pediatric Dentistry, Shanghai Ninth People’s Hospital, Shanghai Jiaotong University School of Medicine, No. 639, Zhi Zao Ju Road, Shanghai, 200011 China; School of Chemical Engineering, East China University of Science and Technology, No. 130, Mei Long Road, Shanghai, 200237 China; National Tissue Engineering Center of China, No. 68, East Jiang Chuan Road, Shanghai, 200241 China

## Abstract

**Background:**

This study was aimed to investigate whether regeneration of periodontal ligament (PDL) like tissue could be promoted by stromal cell-derived factor-1 (SDF1) and bone morphogenetic protein-7 (BMP7) induced cell homing in delayed replantation of avulsed teeth.

**Methods:**

Canine mandibular premolar teeth were first extracted and air-dried for 2 h followed by complete detachment of their PDL tissues. The crown and pulp of the teeth were also removed. Twenty-four roots divided into two groups (n = 12/group) were used for the following in vivo transplantation. The roots of Group A were treated with 17 % EDTA for 24 h to achieve demineralization, and then coated with SDF1 and BMP7 supplemented collagen solution. The roots of Group B were similarly treated except being coated with a pristine collagen solution. The above roots were transplanted in the sockets that formed previously during tooth extraction. At 6 months’ post-operation, PDL-like tissue composed of spindle-shaped cells, capillaries and highly organized collagen fibers was observed in the interstitial space between the avulsed root surface and surrounding alveolar bone in Group A. The neo-fibers inserted deeply and perpendicularly into the cementum and adjacent bone. The periodontium-like characteristics of the neo-tissue was confirmed by immunohistochemical staining for collagen I, fibronectin and osteocalcin.

**Results:**

A high incidence of PDL re-establishment as 42 % was achieved for samples of Group A. However, no PDL-like tissue was found but root ankylosis and replacement resorption as well as inflammatory resorption was observed in the replanted roots of Group B.

**Conclusions:**

It can be confirmed that avulsed teeth could be successfully rescued even in delayed transplantation to avoid dentoalveolar ankylosis or replacement resorption via the current developed cell homing method.

**Electronic supplementary material:**

The online version of this article (doi:10.1186/s12967-015-0719-2) contains supplementary material, which is available to authorized users.

## Background

Tooth avulsion is complete displacement of a tooth from its socket, which is one of the most common but severe case in dental trauma [1]. It renders immediate but severe injury on periodontal ligament (PDL). PDL is a type of soft connective tissue interposed between the root of a tooth and the inner wall of adjacent alveolar socket. Hence, its main role is to fix the tooth in its alveolar socket and attenuate any occlusal loads acting on it. When a tooth is avulsed, all fibers of its PDL are torn. Hence, regeneration or re-establishment of PDL is a paramount prerequisite for the survival of avulsed teeth when they are replanted.

Clinical treatment of an avulsed permanent tooth is to replant it into its original socket as soon as possible. There is strong evidence that immediate replantation of avulsed teeth with an extra-alveolar period of less than 5 min yields the best prognosis [2–4]. Consequently, the avulsed teeth could be saved with their function recovered and aesthetic requirements satisfied. Unfortunately, such kind of success was reported to be occurred only as low as 4 % due to the fact that most cases of replantation were delayed [5–7].

In the delayed case, dentoalveolar ankylosis or replacement resorption after replantation is a frequent complication which further interferes with local dentoalveolar growth, eventually resulting in loss of the traumatized tooth [2, 5, 8–10]. Currently, there is no definite treatment to prevent the occurrence of such failed condition. The main difference between in time and delayed replantation is the vitality of PDL cells which are exposed after tooth avulsion [1, 11]. In their proper station, these cells will catalyze the reproduction of new cells which can differentiate and reinstate the supporting tissue [12]. However, when they were exposed for a prolonged time, they would undergo necrosis due to desiccation or improper storage, and the necrotic condition would be exacerbated with time. As to the PDL cells left in the alveolar bone socket, their differentiation state or regeneration capacity could also be diminished with time since tooth avulsion. That is, the in vivo microenvironment for the delayed transplantation of avulsed teeth would also be unfavorable for its regeneration.

In order to save avulsed teeth when replantation is delayed, research efforts in regenerative medicine have been paid to investigate possible means to regenerate PDL structure using either cell based therapy or activation of endogenous repairing system induced by additional biological cues. It was shown in animal models that cultured PDL cells [13, 14], PDL stem cells [15, 16], bone marrow mesenchymal stem cells (MSCs) [17–20], adipose-derived stem cells [21] and other induced pluripotent stem (iPS) cells [22] could potentially induce the formation of new periodontal tissue after being delivered to defect sites under certain conditions. Interestingly, cultured PDL cells applied in a delayed replanted avulsed tooth also resulted in re-establishment of PDL in beagle dogs [23, 24]. However, many hurdles have retarded clinical translation of cell based therapies for periodontal regeneration, for example, extensive ex vivo cell culture [25]. It is thus imperative to develop an alternative strategy.

It is well established that potentially useful cell populations already exist in the body, and attracting these cells to a desired anatomic site by cell homing to promote endogenous regeneration offers new therapeutic options [26]. Cell homing had been regarded as a transportation process of hematopoietic stem cells (HSCs) from blood vessels by transendothelization and subsequent migration [27]. Recently, the definition was expanded as any vivid recruitment of endogenous cells, including stem/progenitor cells or other types of cells, into an anatomic compartment [27]. Moreover, its effectiveness and safety have been demonstrated for tissue repair or regeneration in diverse medical fields [28–31]. Hence, such strategy is favored for clinical translation [32, 33]. The purpose of this study was to investigate whether regeneration of PDL-like tissue could be promoted by cell homing in delayed replantation of avulsed teeth using canine as an animal model. Stromal cell-derived factor-1 (SDF1) and bone morphogenetic protein-7 (BMP7) were chosen as biological cues to induce the desired cell homing.

SDF1 belongs to the CXC subfamily of chemokines [29] and plays important roles by binding to CXCR4, a G protein-coupled receptor of multiple cell lineages [31]. It often serves as a potent chemoattractant to recruit circulating or resident CXCR4-expressing MSCs which are necessary for specific tissue or organ repair. BMP7 is a potent modulator of osteogenesis and bone cell differentiation. Its effect on periodontal-regenerative treatment was evaluated in bony defects around tooth roots in preclinical studies, and significant improvement on bone and cementum regeneration was achieved [34–36]. The hypothesis of this study is that SDF1 and BMP7 may recruit endogenous cells to root surface of avulsed teeth when no original viable PDL cells were available to simulate the clinical practice that replantation of avulsed teeth was delayed. The homed cells are supposed to re-establish PDL-like tissue, whereas dentoalveolar ankylosis or replacement resorption could then be avoided.

We thus devised the current study. Firstly, SDF1 and BMP7 in a collagen solution were coated on EDTA treated avulsed teeth root. The resulting root was then transplanted into the pre-generated alveolar bone socket in a canine mandible premolar region. The animals were finally sacrificed at 6th month after transplantation, and the specimens were harvested for hematoxylin-eosin (H&E) and immunohistochemical staining. It is believed that the current study could shed light on the development of efficient clinical treatment of accidentally traumatic teeth when they could not be transplanted immediately and were improperly stored.

## Methods

### Treatment of roots from extracted teeth in beagle dogs

All experimental procedures were performed following animal protocols approved by the Animal Care and Experiment Committee of Shanghai Jiao Tong University School of Medicine. The involved procedures have therefore been performed in accordance with the ethical standards laid down in the 1964 Declaration of Helsinki and its later amendments. Animals were housed in the Animal Center of Ninth People’s Hospital Shanghai Jiao Tong University School of Medicine. Three one-year old healthy adult beagle dogs weighing 10–12 kg were used in this study. All operations were performed under general anesthesia with 10 mg/kg ketamine hydrochloride (Gutian Pharmaceutical, Fujian, China). After gingiva was separated, mandibular premolars were luxated with an elevator and extracted with forceps to imitate tooth avulsion. The resulting teeth were kept under air-dried condition for 2 h. PDL tissue was then detached from the surface of extracted teeth by rubbing with wet gauzes. These procedures were designed to imitate clinical conditions of delayed replantation of avulsed teeth. After that, the crown region of the teeth was removed using high-speed diamond burs under 0.9 % cold physiologic saline irrigation to prevent the possibility of tooth loss due to improper occlusal force on the root when it was replanted. Then the double-rooted premolars were split into two parts. The dental pulp was removed thoroughly and the roots were then washed thoroughly with 0.1 M phosphate buffered saline (PBS, pH 7.4). Any fractured roots during the above preparation were excluded from the study.

### Surface coating of roots with SDF1 and BMP7

The above 32 intact roots were randomly and equally divided into two groups. In Group A, the roots were first treated with 17 % EDTA (Sinopharm Chemical Reagent, Shanghai, China) for 24 h to achieve partial demineralization. After that, they were immersed in PBS for another 24 h to remove any residue EDTA molecules, and dried by pumping under reduced pressure. At the same time, SDF1 and BMP7 were both dissolved at a same concentration of 100 ng/mL in a neutralized type I collagen (2 mg/mL) solution (ProSpec, Israel). The above dried roots were autoclaved and then immersed into the SDF1 and BMP7 loaded collagen solution for 15 min. The coated roots were then stabilized at 37 °C for 1 h to ensure sufficient gelation as reported previously [31]. In Group B, the roots were similarly treated as those of Group A except that a pristine collagen solution without SDF1 and BMP7 was used for coating.

Four roots of each group were subjected to scanning electron microscope (SEM) observation and the left were for in vivo replantation. Briefly, the samples were pre-fixed in 2 % glutaraldehyde for 2 h at 4 °C, washed twice with PBS at 4 °C, and post-fixed in 1 % osmic acid for 2 h at 4 °C. After three rinses with PBS, the samples were dehydrated through a graded series of ethanol and dried using a critical point (HCP-2, Hitachi, Japan). After that, samples were sputter-coated with gold and images were taken by SEM (Hitachi S-520, Japan) at 15 kV.

### In vivo orthotopic replantation of coated roots

The alveolar bone sockets were created from the premolar region on each side of the lower mandible by removing blood clot and epithelium in the previous socket formed during the tooth extraction under general anesthesia. The autologous roots of different groups were replanted and submerged in the sockets using sterile forceps. The flaps were repositioned over the replanted roots and sewed up with 4–0 silk suture to fix the roots. Totally, 4 roots were replanted in each side of the lower mandible in each dog (n = 12 for each group). The dogs were fed up with a soft diet after the operation in order to reduce potential mechanical interference.

### Samples harvest and specimen processing

At 6 months’ post-transplantation, the animals were humanely sacrificed with an overdose of intravenous sodium pentobarbital at 100 mg/kg body weight. The replanted roots and their surrounding bone were removed from the jaw. The specimens were fixed in 4 % paraformaldehyde in PBS and decalcified in 10 % EDTA for 3 months. The specimens were then dehydrated in ethanol, embedded in paraffin. Subsequently, step-serial sections were made in 4 μm thickness perpendicularly to the long axes of roots.

### Histomorphometric analysis

Three typical sectioning levels were applied to process histological sections: the apical, middle and cervical regions. As to the apical and cervical regions, around 1 mm distance to the end was left before obtaining the first section for histomorphometric analysis. At each sectioning level, three sections at 200 μm intervals were obtained for H&E staining. Histological analysis was done independently by two examiners who are blind for the groups under optical microscope (Olympus BX51, Japan). A grid with four 45° angle intersecting lines was superimposed onto the images of the sections magnified at forty times. The center of the grid coincided with the center of the root canal [37]. Based on the healing condition of the eight points where the radii intersected with the outer circumference of the root surfaces of three sections, periodontal healing pattern was registered as regeneration sites, or resorption sites (inflammatory root resorption or replacement resorption) using modified Andreasen method [37, 38]. The evaluation standard for regeneration sites: periodontal connective tissue was seen interposing between the intact or repaired cementum and bone; inflammatory root resorption: root dentin was resorbed by monoclear or multinuclear cells, with the adjacent connective tissue showing intense inflammation; replacement resorption: alveolar bone was apposed on the root surface. In addition to the above three standards, sites with the following conditions simultaneously were left unclaimed (neither as a regeneration site nor as a resorption site): (1) The structure of dentine and cementum remained intact without any evidence of resorption, but no obvious connective tissue was arranged between cementum and alveolar bone; (2) Interstitial space existed between the root surface and the adjacent alveolar bone. The occurrence of regeneration sites and resorption sites were counted in the three sections of each sample in each group. If 24 points of the three sections obtained from the three sectioning levels of one sample were all registered as regeneration sites, The root could be classified as complete healing if the 24 points were all registered as regeneration sites. The percentage occurrence of complete healing in each group was calculated (Additional file 1: Table S1). Student’s *t* test was used to compare the percentage of regeneration/resorption sites over the total sites between two groups and the significance level was set at *p* < 0.05 (Additional file 1: Table S1; Additional file 2: Figure S1).

### Immunohistochemical staining of regenerated PDL-like tissue

Immunohistochemical analysis was performed using an EnVision™ + kit (DAKO, USA). After the sections were deparaffinized with xylene and dehydrated with ethanol, they were placed in 10 mM citrate buffer (pH 6.0) and heated in a microwave at 700 W for 5 min for the retrieval of antigens in the specimens. Endogenous peroxidase activity was blocked by incubation of the slides in 3 % H_2_O_2_ at room temperature for 10 min. The sections were then incubated with monoclonal antibodies against collagen type I, fibronectin and osteocalcin (R&D Systems, Minneapolis, MN, USA) overnight at 4 °C, followed by incubation with 50 μL of polymer enhancer for 20 min and 50 μL of polymerized HRP-anti mouse IgG for 30 min. The reaction products were visualized with diaminobenzidine (DAB Kit, Maixin Biological, Fuzhou, China), and sections were counterstained with hematoxylin, and evaluated under light microscope. Tris-buffered saline solution was used instead of the primary antibody for negative controls.

## Results

### Morphology of EDTA treated and cytokines coated roots

**Fig. 1:**
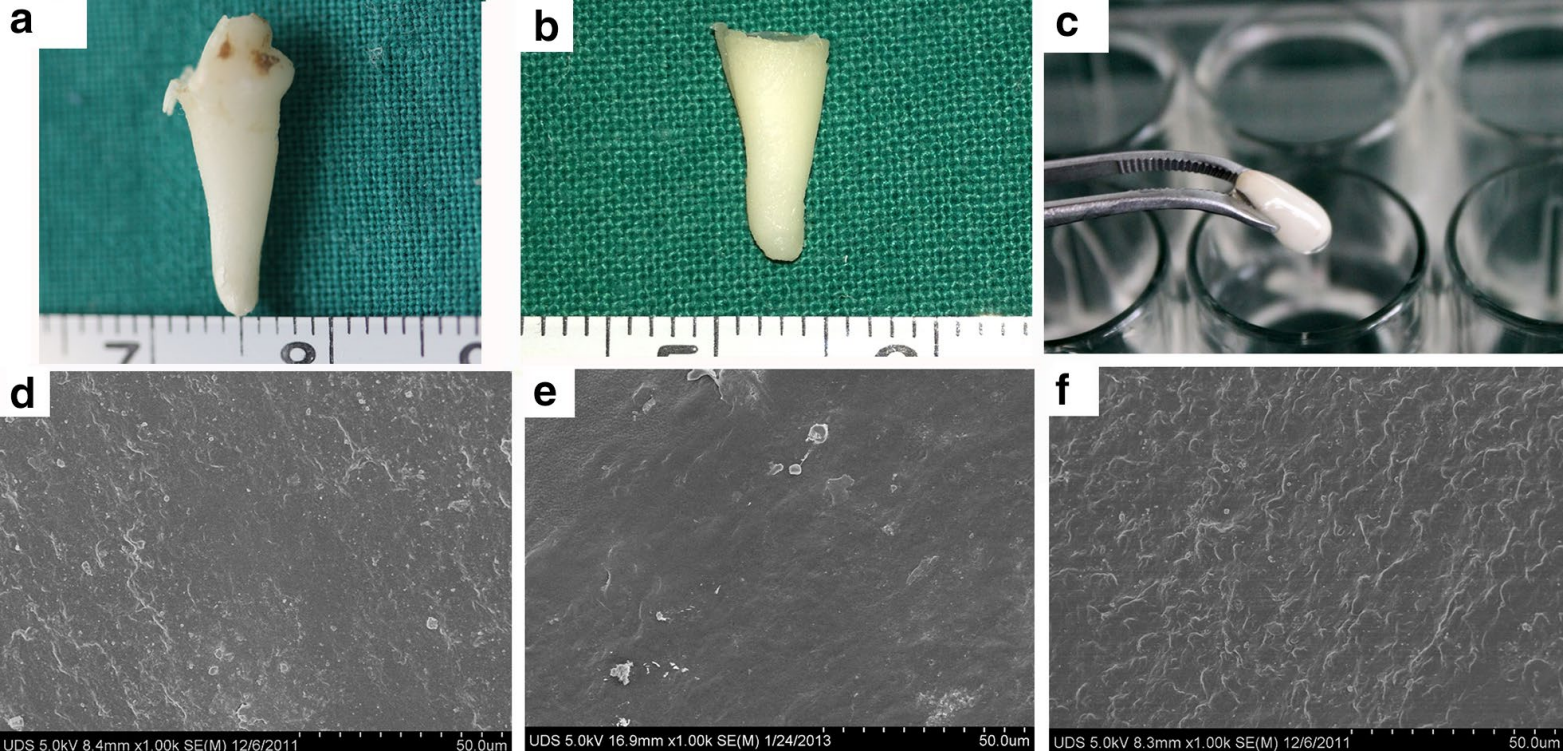
Roots preparation from the extracted teeth of beagle dogs and surface morphology observation of the root using SEM. **a** The tooth was first extracted from the beagle dog and then air-dried for 2 days. The PDL tissue was then detached from the surface of extracted tooth with a wet gauze. **b** After that, the crown region and dental pulp of the teeth was removed thoroughly. The resulting roots were EDTA treated for 24 h, followed by washing and drying. **c** The roots of Group A were immersed in the SDF1 (100 ng/ml) and BMP7 (100 ng/ml) loaded collagen solution for 15 min. Surface morphology of the root before (**d**) and after (**e**) EDTA treatment as well as after (**f**) being coated with SDF1 and BMP7 supplemented collagen solution was observed by scanning electron microscopy (SEM)

Gross views of the extracted tooth after PDL detachment and the root after EDTA treatment as well as the cytokines coated root of Group A were shown in Fig. 1a, b, c, respectively. According to SEM observation, the root surface before EDTA treatment was compact without any obvious pores as shown in Fig. 1d. Nodule-featured surface morphology was observed in roots after EDTA treatment (Fig. 1e). However, after cytokines coating (Fig. 1f), it seems that such micro-morphology was covered and could not be seen clearly. This is also the condition for samples of Group B (results not shown).

### Histological observation

**Fig. 2:**
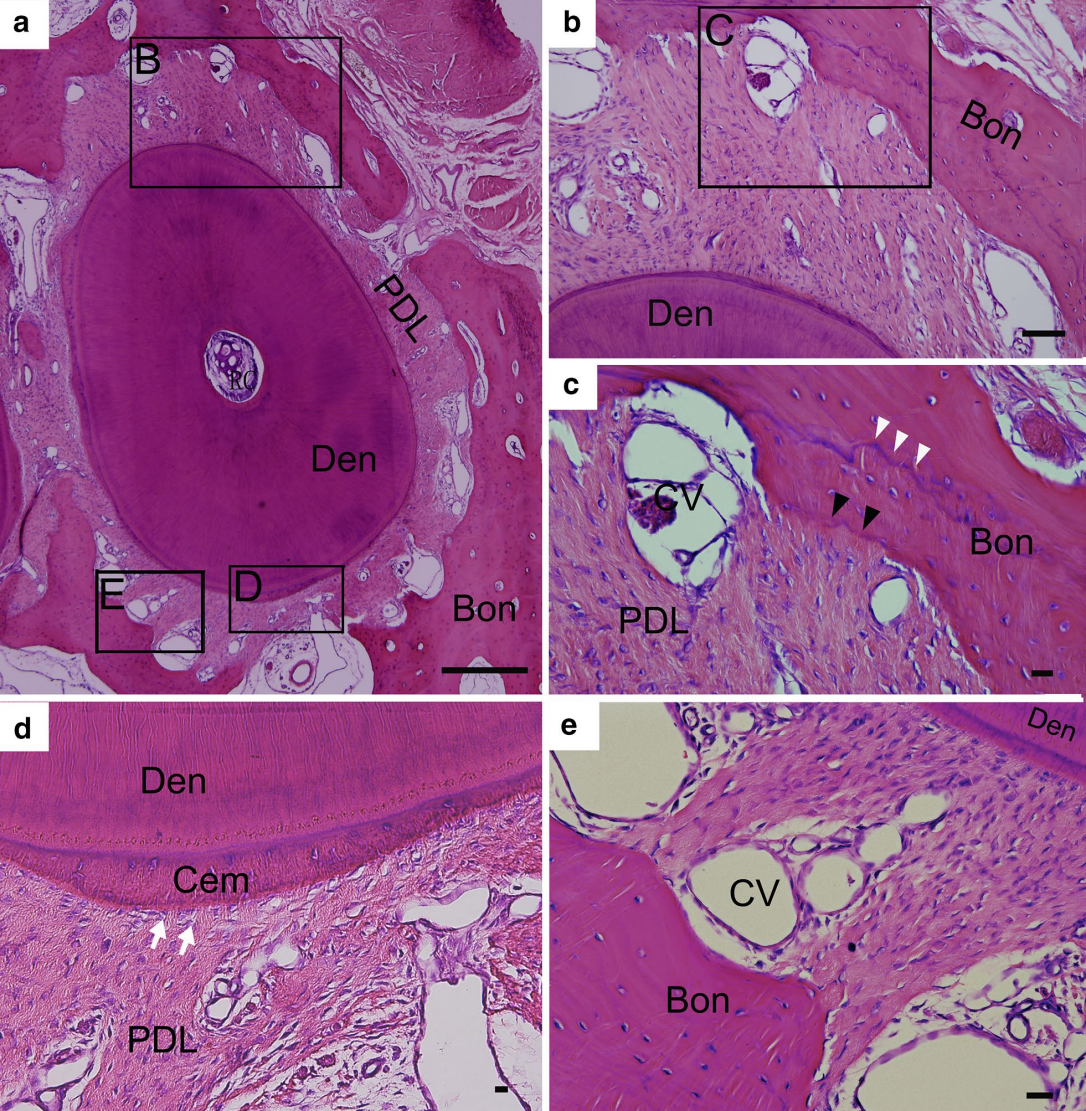
Histological observation of the SDF1 and BMP7 coated roots after being implanted orthotopically in canine alveolar bone socket for 6 months. **a** The gross histological morphology of one root of Group A. At 6 months after replantation, the interstitial space between alveolar bone and the replanted root were already filled with a high content of newly formed PDL-like connective tissue. The newly formed bundles of dense collagen fibers interposed between the cementum on the root surface and the adjacent alveolar bone. **b**, **d**, **e** are high-magnification images of the *lined rectangular areas* in a, while **c** is high-magnification image of the *lined rectangular area* in **b**. **b**, **c**, **e** from the localized area, it can be seen that bundles of collagen fibers inserted perpendicularly into the surrounding alveolar bone indicated by *black arrow heads*. **c** Growth and progression of alveolar bone towards the neo-connective tissue could be ascertained, as layered structure indicated by *white arrow heads* in the interface was observed. **d** The neo-collagen fibers were observed interpenetrating into the cementum as indicated by *white arrows*. Angiogenesis was also enhanced as rich capillary vessel structures were distributed in the neo-connective tissue (**b**–**e**). *Bon* alveolar bone, *Cem* cementum, *Den* dentin, *PDL* periodontal ligament, *CV* capillary vessel, *RC* root canal. *Bar scales* 250 μm for **a**–**e**

All the dogs recovered very well for 6 months postoperatively before they were euthanized for histological examination. Figure 2a shows the histological morphology of one sample of Group A. It can be seen that at 6 months after replantation the interstitial space between alveolar bone and the replanted root were already filled with a high content of newly formed PDL-like connective tissue. The PDL-like tissue was composed of spindle-shaped cells lined with dense collagen fibers and capillaries (Fig. 2b–e). The newly formed bundles of collagen fibers interposed between the cementum on the root surface and the adjacent alveolar bone. From the localized area (Fig. 2b, c, e), it can be seen that collagen fibers inserted perpendicularly into the surrounding alveolar bone indicated by black arrow heads. Layered structure of the alveolar bone was observed as indicated by white arrow heads in the interface in Fig. 2c. On the other hand, the neo-collagen fibers were observed interpenetrating into the cementum (white arrows in Fig. 2d). Angiogenesis was also enhanced as rich capillary vessel structures (briefed as CV in Fig. 2c, e) were distributed in the neo-connective tissue. The above root shown in Fig. 2 is an example of complete healing. Among the 12 replanted roots in Group A, 5 roots exhibited such kind of complete healing accounting for 42 % (Additional file 1: Table S1). Detailed histomorphometric analysis of all the transplanted roots was shown in Additional file 1: Table S1. The periodontal healing pattern was addressed by counting the 24 points of three histological sections according to the method described in the experimental part. However, inflammatory resorption or dentoalveolar ankylosis did occur occasionally in some points of the rest 7 roots.

**Fig. 3:**
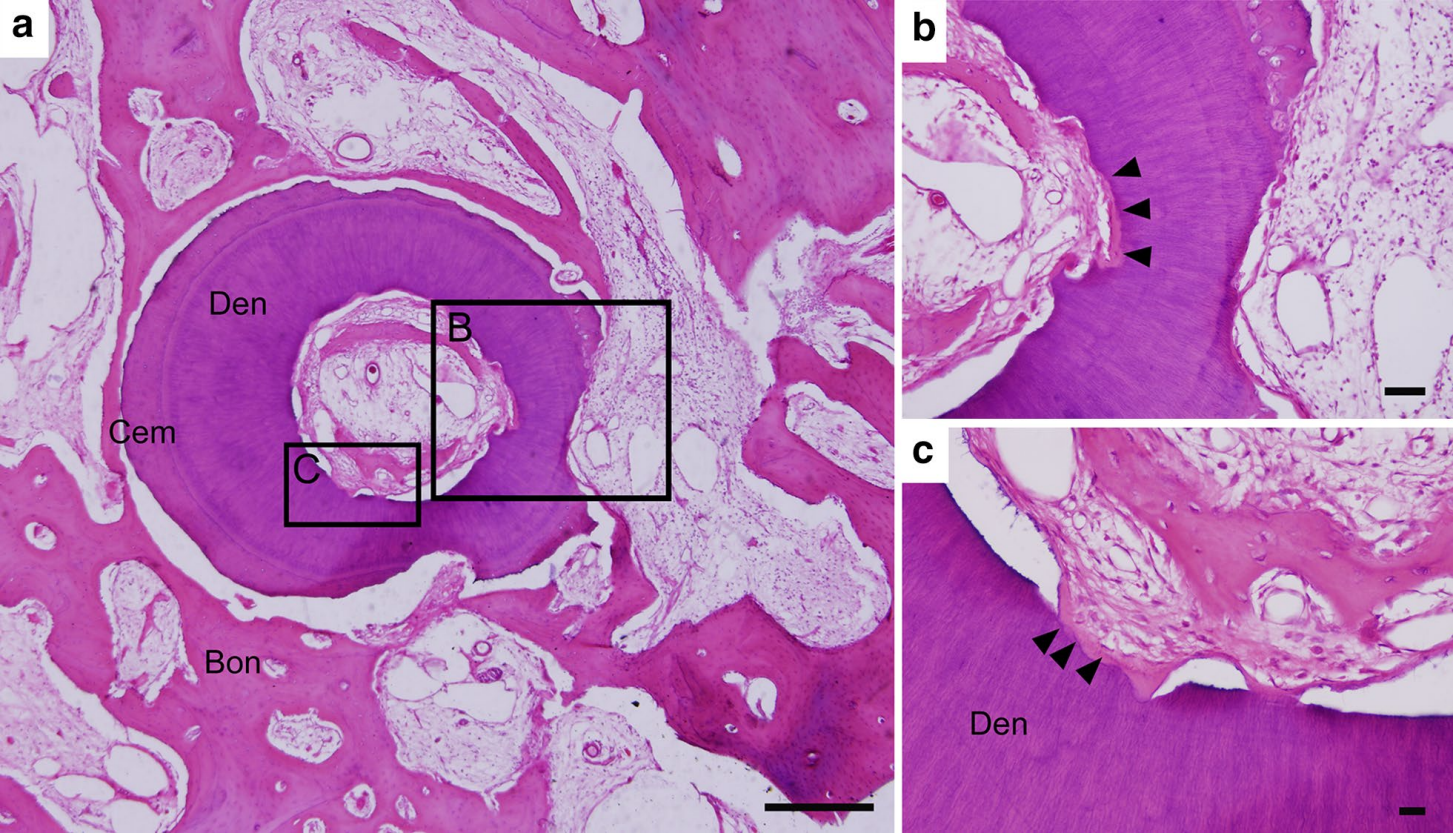
A typical case of the root in Group A with occurrence of replacement resorption and inflammatory root resorption. **a** The overall histological morphology of the root exhibited the progress of both replacement resorption (dentoalveolar ankylosis) and inflammatory resorption on several sites of the root surface. **b** and **c** are high-magnification images of the *lined rectangular areas* in **a** dentoalveolar ankylosis or root replacement resorption was observed in both the inner dentine wall and the outer dentine/cementum structure of the replanted root as indicated by *black arrow heads*. Inflammatory root resorption was also observed in some local areas of the root surface (**b**). Although most of the dentine structure was remained, aggressive root resorption already occurred as some of the surface of the implanted root was covered by alveolar bone. In such case, PDL like tissue could not be observed. *Bon* alveolar bone, *Cem* cementum, *Den* dentin. *Bar scales* 250 μm for **a**–**c**

Figure 3 shows a typical case of the roots in Group A with occurrence of inflammatory resorption and dentoalveolar ankylosis. Inflammatory root resorption could be located in some area of the root surface where fibrous soft tissue with a high density of inflammatory cells was observed (Fig. 3a, b). In areas with ankylosis, the resorbed root surface was in direct contact with the alveolar bone as shown in Fig. 3b, c (indicated by black arrow heads). Dentoalveolar ankylosis occurred both from the inner dentine wall and the outer dentine/cementum structure of the replanted root. Some of the replanted root surface was covered by alveolar bone, although such sample still remained most of its dentine and cementum structure according to the histological images (Fig. 3a). Unfortunately, a high incidence of such kind of resorption in varying extents happened as shown in Additional file 1: Table S1. The average percentage of regeneration sites over the total sites of Group A was 77.08 %, and the respective average percentage of resorption sites over the total sites was significantly lower [14.58 %, *p* < 0.01 (Additional file 1: Table S1; Additional file 3: Figure S2)].

**Fig. 4:**
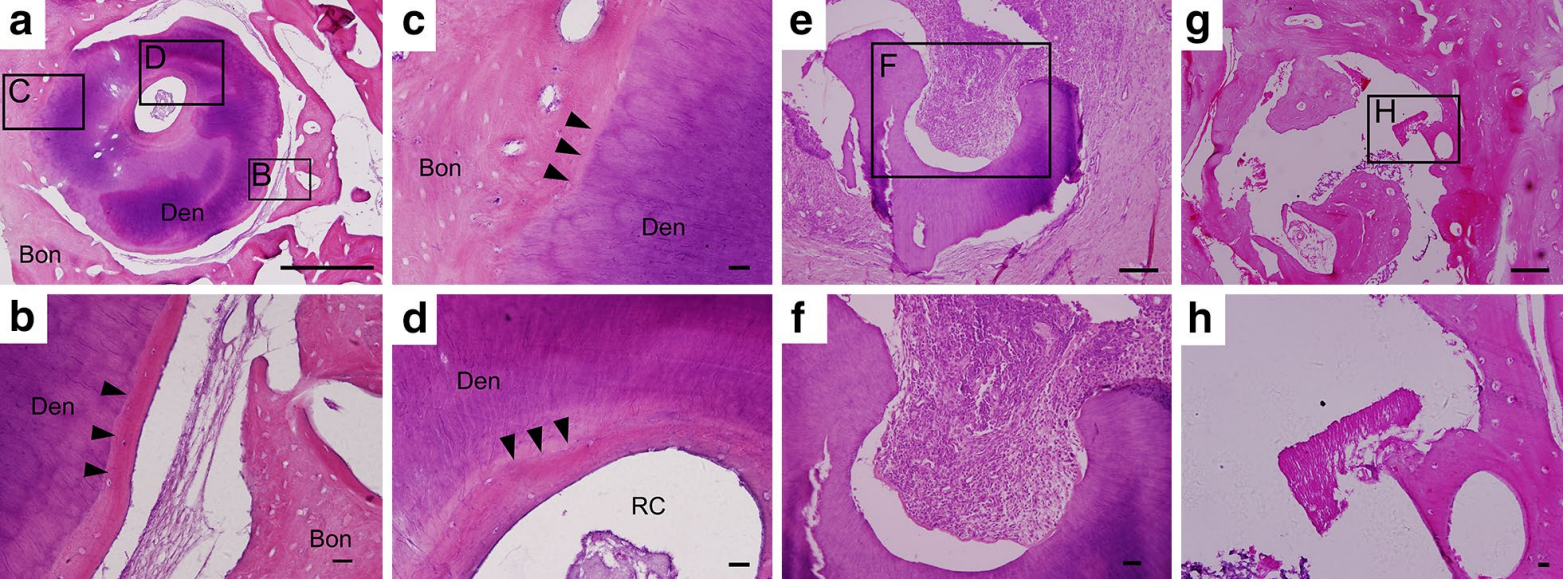
Histological morphology of three typical cases of the roots in Group B. **a**, **e**, **g** are the overall histological morphology of the roots, and **b**–**d**, **f**, **h** are magnified images of the *lined rectangles* in **a**, **e**, and **g**, respectively. **a**–**d** is a typical case of root replacement resorption. **c** The root surface was in direct contact with the alveolar bone, characteristic of dentoalveolar ankylosis. It seems that the tubular structure of dentine was underwent vivid degradation. In some regions of dentine, the original compact structure became loose with the appearance of a few pores. Layered pattern could be seen in the dentin structure. Some of the dentinal tubules were distorted, while some were already replaced by alveolar bone structure (**b**, **d**). **e** and **f** is a typical case of inflammatory root resorption. Intensive inflammation was observed with the presence of accumulation of numerous inflammation cells around the surface of the root. The root structure was seriously destroyed and only a small incomplete structure was *left*. **g** and **h** is a case which may be subjected to both replacement resorption and inflammation resorption. A few dentinal tubules debris could be identified and the whole root could not be found anymore. Formation of dentoalveolar structure was observed in some areas surrounding the dentinal debris, whereas others were lacunae structure. *Bon* alveolar bone, *Cem* cementum, *Den* dentin, *RC* root canal. *Bar scales* 250 μm for **a**–**h**

In roots of Group B, no complete healing was obtained. Replacement resorption or inflammation resorption, or both were occurred on all of them. Figure 4 shows the histological morphology of three typical cases of the roots in Group B. Figure 4a–d is a typical case of root replacement resorption. Some part of the root surface was in direct contact with the surrounding alveolar bone, characteristic of dentoalveolar ankylosis (indicated by black arrow heads, Fig. 4a, c). Although most of the interstitial space between alveolar bone and the replanted root was remained empty, most of the root surface was covered by a thin layer of alveolar bone-like tissue (Fig. 4b). In some regions of dentine, the original compact structure became loose with the appearance of quite a few pores (Fig. 4a). Layered pattern was also seen in the dentin structure (Fig. 4a). Some of the dentinal tubules were distorted, while some were already replaced by alveolar bone structure (Fig. 4b, d). Figure 4e and f is a typical case of inflammatory root resorption in Group B. Intensive inflammation was observed with the accumulation of numerous inflammation cells around the surface of the root. The root structure was destroyed with only a small portion left. Furthermore, in some cases of Group B, the roots were almost gone as shown in Fig. 4g, h. Only a few dentinal tubules debris could be identified. Formation of dentoalveolar structure was observed in some areas surrounding the dentinal debris, whereas others were lacunae structure. Most probably, it could be a case which was subjected to both replacement resorption and inflammation resorption. As to the samples of Fig. 4e, g, grossly, only some relevant tissue could be harvested for H&E processing. The average percentage of resorption sites over the total sites in Group B was significantly higher than that in Group A [82.99 versus 14.58 %, *p* < 0.01 (Additional file 1: Table S1)].

### Immunohistochemical observation of regenerated PDL-like tissue

**Fig. 5:**
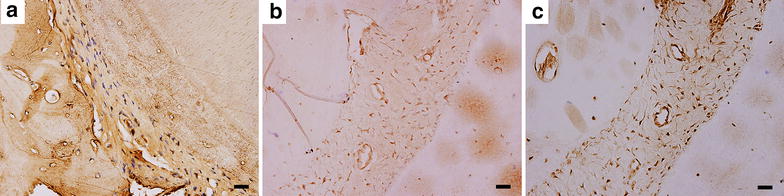
Immunohistochemical staining of regenerated PDL-like tissue in Group A. Immunohistochemical staining for collagen I (**a**), fibronectin (**b**), and osteocalcin (**c**) of the regenerated PDL-like tissue. The periodontium-like characteristics of the neo-tissue could be confirmed by positive immunohistochemical staining for collagen type I, fibronectin and osteocalcin. *Bar scales* 100 μm for **a**–**c**

The newly formed PDL-like tissue in Group A was further subjected to immuno-histological staining to detect the expression of characteristic proteins of connective tissue, such as collagen I and fibronectin (Fig. 5a, b). The osteogenic ability of cells in the newborn tissues was evaluated by detecting the expression of osteocalcin (Fig. 5c). Positive staining for collagen type I was obviously observed in newly formed connective tissue, cememtum and adjacent alveolar bone (Fig. 5a). In the PDL-like tissue, there were deep stained thick fibers spreading asymmetrically (Fig. 5a). Fibronectin was also expressed strongly in newborn PDL-like tissue, whereas the adjacent bone did not express any fibronectin (Fig. 5b). Moreover, strong staining of osteocalcin was seen in the cells of newborn tissue (Fig. 5c). Hence, the periodontium-like characteristics of the neo-tissue was confirmed by positive immunohistochemical staining for collagen type I, fibronectin and osteocalcin.

## Discussion

In the present study, a PDL-like neo-tissue could be successfully regenerated between the replanted root surface and its surrounding alveolar bone induced by surface coating of the root with SDF1 and BMP7. Its highly organized collagen fibers inserted perpendicularly and deeply into the cementum and adjacent bone. The periodontium-like characteristics of neo-tissue could be ascertained based on the histological morphology and positive immunohistological staining for collagen I, fibronectin and osteocalcin. As a soft connective tissue which interposed tightly and continuously between the root and the inner wall of adjacent alveolar socket, it could further take its duty to fix the tooth and attenuate any occlusal loads acting on it. That is, the integrity of periodontal structure was restored. Since the replanted root surface was stored in an air-dried condition for 2 h followed by PDL removal as well as demineralization, it can be ensured that no viable PDL cells were present. Because it was demonstrated that if the extra-oral dry time of an avulsed tooth is more than 60 min, all PDL cells including the stem/progenitor cells residing in the area on the root surface are nonviable [39]. Hence, the recruitment of endogenous cells to re-establish PDL-like tissue was mediated as expected by SDF1 and BMP7. The efficacy of the current cell homing method could then be ascertained to rescue avulsed teeth when they were given up clinically.

Endogenous stem/progenitor cells residing in many adult organs/tissues or circulating in the blood are normally termed ‘tissue-specific stem cells’ [40]. These cells are usually hosted in specific niches where there is a broad spectrum of extracellular substrates or growth factors that regulate stem cell survival, self-renewal, and differentiation. This is a natural programme designed for tissue maintenance and self-repair. In case of disease or injury, the endogenous stem/progenitor cells would migrate to and engraft in the affected tissue, then proliferate extensively and differentiate specifically. For example, bone marrow-derived MSCs would constantly leave the bone marrow and enter tissue-specific niches, while replenish themselves simultaneously to maintain their original balance [41]. It was also reported that both local MSCs derived from the injured tissue and circulating MSCs collaborated to heal the damaged organ [42]. However, when such innate reparative or self-repair system is not sufficient, it is necessary to introduce additional signals which would instruct the recruitment of more endogenous stem cells to the target site and activation of regeneration [28, 31, 43, 44]. Such strategy was proven to be promising for clinical translation because it avoids extensive ex vivo cell culture and sometimes minimizes the number and invasiveness of associated clinical procedures. However, the success of the above desired cell homing strategy depends on first identifying the bioactive macromolecules that mediate organ-specific homing and then manipulating these molecules’ activity to catalyze the homing process.

In our study, it was demonstrated that SDF1 and BMP7 had the ability to direct the navigation of stem cells to the interstitial space between replanted root and surrounding adjacent alveolar bone, thereby establishing the integrated PDL like structure. SDF1 is a cytokine that belongs to the CXC class of chemokine proteins. It is the only known natural chemokine in the body that is activated by binding to CXCR4 receptors which are expressed by HSCs, endothelial progenitor cells, MSCs and the like [28, 45]. The SDF1/CXCR4 complex was shown to play a key role in stem cell homing. When in damaged tissues, the increase in local SDF1 is a key factor that regulates stem cell homing and recruitment to injured tissues [28, 31, 45–51]. Results were also shown that intravenous injection of SDF1 promoted the mobilization of HSCs [28, 49–51]. Most probably, SDF1 in the current work had homed a considerable amount of mesenchymal and endothelial stem/progenitor cells from native alveolar bone into the surface of EDTA treated roots that were replanted in canine jaw bone [52–54]. BMP 7 was chosen for its reported effects on dental pulp cells, fibroblasts and osteoblasts in elaborating mineralization [55–57]. Its role has been elaborated as the induction of multiple transcription osteogenic/odontogenic genes during osteoblast differentiation and phosphorylation [58, 59]. Hence, BMP7 may be responsible highly for the newly formed mineralized tissue in the current canine alveolar socket.

Kim et al. had carried out pioneering works on PDL tissue regeneration via cell homing mediated by SDF1 and BMP7 [31]. An artificial scaffold made from a hybrid of poly-ε-caprolactone and hydroxyapatite was used in their work. As to the in vivo model, in their case, alveolar bone socket made from the extraction of an incisor was immediately used to transplant artificial prepared scaffold. However, for avulsed teeth, the original sockets would usually be left for a prolonged time, e.g. more than 2 days in the current work, before replantation. Hence, whether such cell homing method could be used to save avulsed teeth when no viable PDL cells were left on root surface, and how to apply SDF1 and BMP7 in traumatic tooth model occurred frequently in clinic remained to be answered. To do this, EDTA treatment for 24 h of avulsed roots which were first air-dried for 2 h was carried out in order to achieve demineralization. It was reported that the demineralization treatment of the root surface could help to expose collagen fibers on root cementum and promote a contact surface for reattachment of regenerated PDL collagen fibers [60]. Moreover, the increase in the surface roughness with EDTA treatment was also proposed to promote the adherence of coated cytokines and thereby their favored release. The release of inherent proteins or growth factors within cementum could also be enhanced via demineralization. Therefore, it was supposed necessary for the function of SDF1 and BMP7 to finally re-establish the PDL-like structure layed down by the recruited endogenous cells. As shown in the histological morphology of the experimental sample, the highly organized collagen fibers of PDL-like tissue inserted perpendicularly into the cementum.

The present study is the first to demonstrate that PDL-like tissue could be regenerated via SDF1 and BMP7 mediated cell homing in avulsed teeth of delayed replantation. However, to further pave the way for dental clinical application of such cell homing strategy, quite a few issues still remain to be addressed. First, the individual role of SDF1 and BMP7 in the current research model, respectively, was not identified. Second, the valid dosage of SDF1 and BMP7, that is their respective amounts being coated on the root surface, remains to be determined after the verification of current hypothesis. Third, the optimal EDTA treatment duration that would yield most favorable condition for collagen fibers re-attachment was to be optimized. Fourth, the success rate addressed as complete healing is quite low and even less than 50 %. One important cause of such low efficiency was probably due to is the fact that our collagen coating method might not be the optimal option for the delivery of SDF1 and BMP7 with a slow release, as it is well known that the release kinetics of growth factors is very important for the therapeutic efficiency. Finally, whether the source of the engrafted cells could be traced with the determination of cell identity is also important for further development. These issues are focus of our ongoing work.

## Conclusion

In conclusion, avulsed teeth could be successfully rescued even in delayed transplantation to avoid dentoalveolar ankylosis or replacement resorption via the current developed cell homing method. The combination of EDTA root conditioning and SDF1 and BMP7 coating promote PDL regeneration whereas the EDTA root conditioning alone is not able to sustain such regeneration. A PDL-like neo-connective tissue was reestablished with its highly organized collagen fibers interposing tightly and perpendicularly between the replanted root surface and its surrounding alveolar bone. This study could further pave the way toward clinical translation of PDL regeneration.

## Additional files

10.1186/s12967-015-0719-2 Comparison of periodontal healing pattern for two groups.
10.1186/s12967-015-0719-2 The method of modified Andreasen. The eight points of every section where the radii intersected with outer outer circumference of the root surfaces were analyzed. The center of the grid coincided with the center of the root canal [3]. Based on the healing condition of the points, periodontal healing pattern was registered as regeneration sites, or resorption sites (inflammatory root resorption or replacement resorption) using modified Andreasen method [3,4]. Regeneration sites: periodontal connective tissue was seen interposing between the intact or repaired cementum and bone; inflammatory root resorption: root dentin was resorbed by monoclear or multinuclear cells, with the adjacent connective tissue showing intense inflammation; replacement resorption: alveolar bone was apposed on the root surface. In addition to the above three standards, sites with the following conditions simultaneously were left unclaimed (neither as a regeneration site nor as a resorption site): 1. The structure of dentine and cementum remained intact without any evidence of resorption, but no obvious connective tissue was arranged between cementum and alveolar bone; 2. Interstitial space existed between the root surface and the adjacent alveolar bone. The occurrence of regeneration sites and resorption sites were counted in the three sections of each sample in each group. If 24 points of the three sections obtained from the three sectioning levels of one sample were all registered as regeneration sites, the root could be classified as complete healing if the 24 points were all registered as regeneration sites. (RC: root canal).
10.1186/s12967-015-0719-2 Masson staining of roots. a, b: regeneration sites, periodontal connective tissue was seen interposing between the intact or repaired cementum and bone, The newly formed bundles of collagen fibers interposed between the cementum on the root surface and the adjacent alveolar bone. From the localized area(indicated by black arrow heads); c-f: inflammatory root resorption: intensive inflammation was observed with the accumulation of numerous inflammation cells around the surface of the root(c, d); the root structure was almost gone and only a few dentinal tubules debris could be identified.(e, f). a, c, e: 10× ; b, d, f: 20× . (PDL: periodontal ligament; Den: dentin; Cem: cement).
