# Supplementary material for: PDL regeneration via cell homing in delayed replantation of avulsed teeth
Source: J Transl Med. 2015 Nov 14;13:357. doi: 10.1186/s12967-015-0719-2 (PMC4647325; doi:10.1186/s12967-015-0719-2)
Supplement: Supplementary file 1 — 10.1186/s12967-015-0719-2 Comparison of periodontal healing pattern for two groups. [file 12967_2015_719_MOESM1_ESM.docx]

Supplement File:

sTable1. Comparison of periodontal healing pattern for two groups

| **Groups** | **Complete healing** | **Regeneration** | **Resorption** |
| --- | --- | --- | --- |
|  | **(%)** | **‾x(%) ± sd** | **‾x(%) ± sd** |
| **A(n=12)** | 42% | 77.08±26.80^*#^ | 14.58±23.93^*^ |
| **B(n=12)** | 0 | 0 | 82.99±30.90 |

n=the number of teeth examined in each group.Student’s t-test was used to compare the percentage of regeneration/resorption sites over the total sites between two groups and the significance level was set at p<0.05.

‾x(%)=the mean value of percentages of regeneration/resorption sites over the total 24 sites of 12 samples in each group.

Sd=standard deviation.

^*^p<0.01 in comparison to Group B.

^#^P<0.01 in comparison to the mean value of percentages of resorption sites over the total 24 sites in the same group.
