# Supplementary figures and images for: PDL regeneration via cell homing in delayed replantation of avulsed teeth
Source: J Transl Med. 2015 Nov 14;13:357. doi: 10.1186/s12967-015-0719-2 (PMC4647325; doi:10.1186/s12967-015-0719-2)

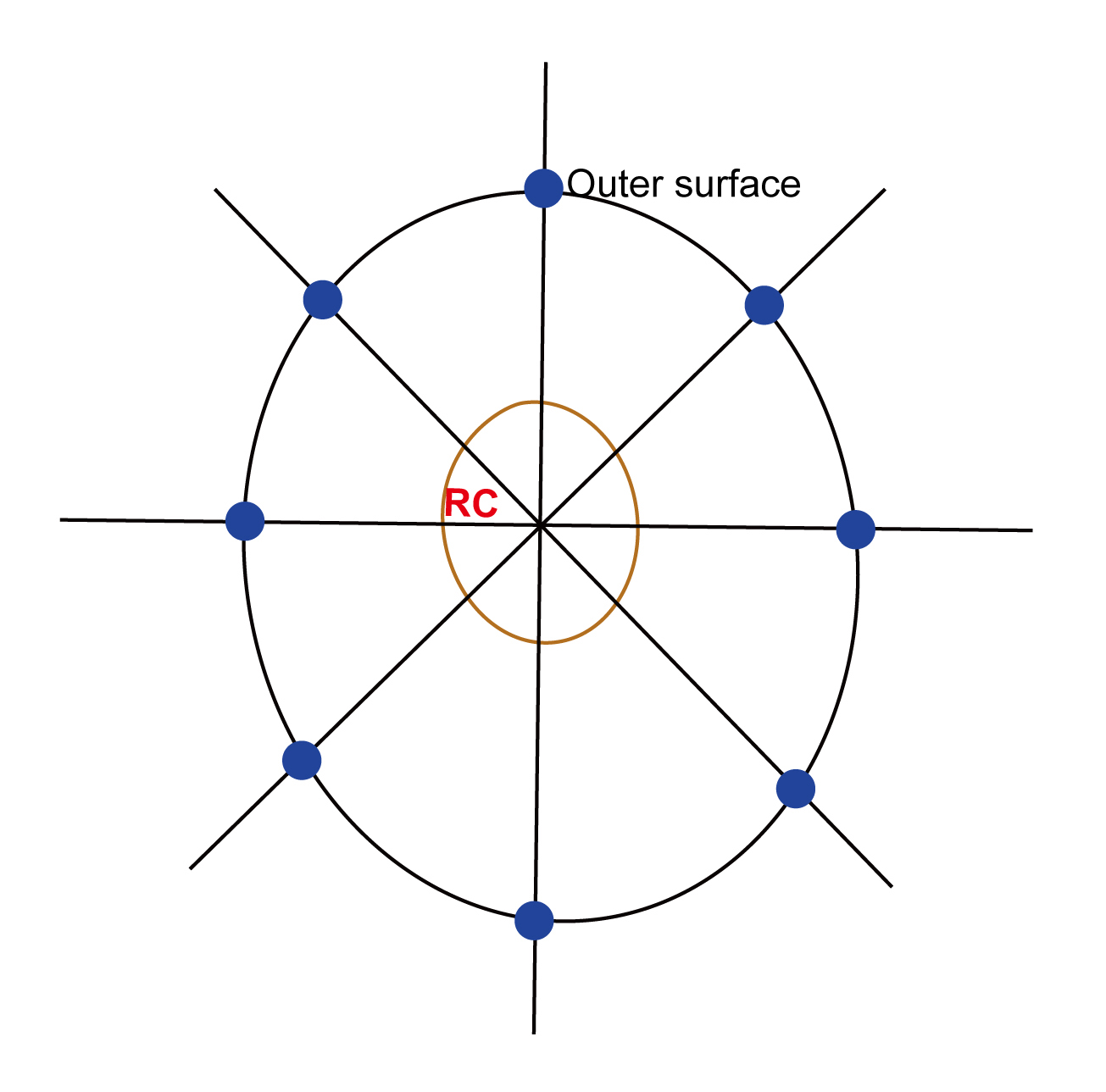

Supplement: Supplementary file 2 — 10.1186/s12967-015-0719-2 The method of modified Andreasen. The eight points of every section where the radii intersected with outer outer circumference of the root surfaces were analyzed. The center of the grid coincided with the center of the root canal [3]. Based on the healing condition of the points, periodontal healing pattern was registered as regeneration sites, or resorption sites (inflammatory root resorption or replacement resorption) using modified Andreasen method [3,4]. Regeneration sites: periodontal connective tissue was seen interposing between the intact or repaired cementum and bone; inflammatory root resorption: root dentin was resorbed by monoclear or multinuclear cells, with the adjacent connective tissue showing intense inflammation; replacement resorption: alveolar bone was apposed on the root surface. In addition to the above three standards, sites with the following conditions simultaneously were left unclaimed (neither as a regeneration site nor as a resorption site): 1. The structure of dentine and cementum remained intact without any evidence of resorption, but no obvious connective tissue was arranged between cementum and alveolar bone; 2. Interstitial space existed between the root surface and the adjacent alveolar bone. The occurrence of regeneration sites and resorption sites were counted in the three sections of each sample in each group. If 24 points of the three sections obtained from the three sectioning levels of one sample were all registered as regeneration sites, the root could be classified as complete healing if the 24 points were all registered as regeneration sites. (RC: root canal). [file 12967_2015_719_MOESM2_ESM.jpg]

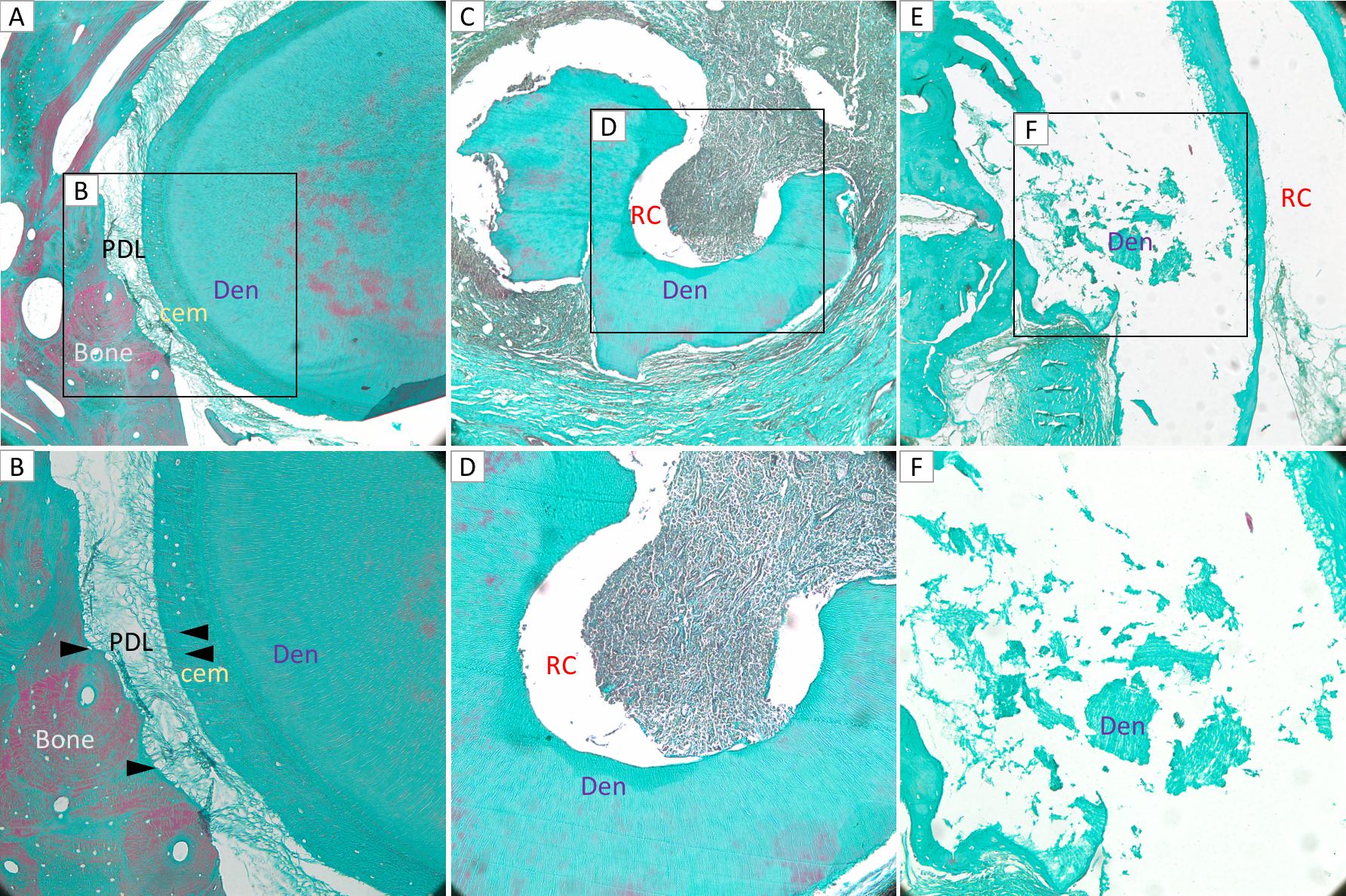

Supplement: Supplementary file 3 — 10.1186/s12967-015-0719-2 Masson staining of roots. a, b: regeneration sites, periodontal connective tissue was seen interposing between the intact or repaired cementum and bone, The newly formed bundles of collagen fibers interposed between the cementum on the root surface and the adjacent alveolar bone. From the localized area(indicated by black arrow heads); c-f: inflammatory root resorption: intensive inflammation was observed with the accumulation of numerous inflammation cells around the surface of the root(c, d); the root structure was almost gone and only a few dentinal tubules debris could be identified.(e, f). a, c, e: 10× ; b, d, f: 20× . (PDL: periodontal ligament; Den: dentin; Cem: cement). [file 12967_2015_719_MOESM3_ESM.jpg]
